# Establishment and characterization of new cell lines of anaplastic pancreatic cancer, which is a rare malignancy: OCUP-A1 and OCUP-A2

**DOI:** 10.1186/s12885-016-2297-y

**Published:** 2016-04-11

**Authors:** Kotaro Miura, Kenjiro Kimura, Ryosuke Amano, Sadaaki Yamazoe, Go Ohira, Akihiro Murata, Kohei Nishio, Tsuyoshi Hasegawa, Masakazu Yashiro, Bunzo Nakata, Masaichi Ohira, Kosei Hirakawa

**Affiliations:** Department of Surgical Oncology, Osaka City University Graduate School of Medicine, 4-3, 1-chome, Asahimachi, Abeno-ku, Osaka city, Osaka 545-8585 Japan; Department of Hepato-Biliary Pancreatic Surgery, Osaka City General Medical Center, 13-22, 2-chome, Miyakojimahondori, Miyakojima-ku, Osaka city, Osaka 534-0021 Japan; Department of Microbiology & Molecular Cell Biology, Leroy T. Canoles Jr. Cancer Research Center, Eastern Virginia Medical School, Harry T. Lester Hall 421 651 Colley Avenue,, Norfolk, 23501 VA USA; Department of Surgery, Kashiwara Municipal Hospital, 1-chome, 7-9, Hozenji, Kashiwara city, Osaka 582-0005 Japan

**Keywords:** Anaplastic pancreatic cancer, Human cell line, Rare malignancy

## Abstract

**Background:**

Anaplastic pancreatic cancer (APC) cell lines have been scarcely established.

**Methods:**

The morphology, gene expressions, karyotyping and epithelial-mesenchymal transition markers of newly established APC cell lines OCUP-A1 and OCUP-A2 were analyzed. Their abilities of proliferation under normoxia and hypoxia, migration and invasion were compared to 4 commercially available pancreatic ductal adenocarcinoma (PDA) cell lines. Their induction of angiogenesis, stem-like cell population and subcutaneous tumor growth in nude mice were estimated, comparing 2 PDA cell lines examined here.

**Results:**

OCUP-A1 and OCUP-A2 cells continuously grew with spindle and polygonal shapes, respectively. Gene analysis revealed 9 gene mutations including KRAS and TP53. Karyotyping clarified numerical structural abnormalities in both cells. Loss of E-cadherin and expression of vimentin in both cell lines were observed. The doubling time of both cell lines was approximately 20 h. Proliferation, migration and invasion abilities were not notable compared to other PDA cell lines. However stem-like cell population of both cell lines was superior to a part of PDA cell lines. Moreover OCUP-A1 showed stronger hypoxia tolerance and induction of angiogenesis than other PDA cell lines. The tumorigenicity in vivo of OCUP-A2 was stronger than conventional PDA cell lines.

**Conclusions:**

The OCUP-A1 and OCUP-A2 cell lines of rare malignancies might be useful for investigating the biology of pancreatic cancer.

## Background

Although the number of treatment strategies has been increasing for pancreatic ductal adenocarcinoma (PDA), it still has poor prognosis. The 5-year survival rate is <5 %, and the outcomes have not changed for almost 50 years [1, 2]. Anaplastic pancreatic cancer (APC) is a rare histopathological type of PDA. APC is one of the synonyms for undifferentiated carcinoma [3], and is classified as grade 4 in the World Health Organization classification [4]. In Japan, approximately 0.1 % of all pancreatic cancer was this type [5]. In general, the percentage reported ranged from approximately 2–12 % [6–8]. Several studies have found that APC frequently occurs in patients with an average age of 60 years [8, 9]. The incidence of this tumor is three times higher in men than in women [8]. Common clinical manifestations are abdominal pain, fatigue, and anorexia [8]. Computed tomography (CT) has shown that the tumor is likely to show low density in the central region and high density in the marginal region [3]. These findings suggest that the central area is necrotic, and the marginal area has abundant blood flow [3]. Only surgical resection offers the possibility of a complete cure [3]. Systemic chemotherapy administered for conventional PDA is generally not effective [10]. The overall survival of APC is several months [11]. This is less than seen with conventional PDA [3]. Regarding APC, not much is yet known about its biological features. An effective treatment modality has not been identified for either APC or PDA. In the present study, we succeeded in establishing two cell lines of APC, and characterized the properties that might lead to successful therapy. These cell lines were useful for researching the basic characteristics of pancreatic cancer because the two established cell lines had several features that caused a greater degree of aggressiveness than with conventional PDA.

## Methods

### Patients

#### OCUP-A1

A man in his 60s with abdominal pain was suspected as having pancreatic cancer, as seen on CT. The values of carcinoembryonic antigen (CEA), cancer antigen (CA) 19–9, SPan-1 and DUPAN-2 were 3.1 ng/mL, 4 U/mL, >3 U/mL (reference value), and >25 U/mL (reference value), respectively. The patient underwent distal pancreatectomy with resection of the portal vein which accompanied a tumor embolism. The pathological stage was T3N1M0, UICC stage IIB. He died due to recurrence and rapid progression of the cancer at two months after surgery.

#### OCUP-A2

A man in his 30s with left upper quadrant pain was diagnosed by CT as having a malignant pancreatic neoplasm with liver metastasis. The values of CEA, CA19-9, SPan-1 were 0.7 ng/mL, 394 U/mL and 60 U/mL, respectively. Although the diagnosis was unresectable pancreatic cancer, the patient underwent distal pancreatectomy in order to control the bleeding in his stomach caused by the invading primary tumor. The pathological classification of the cancer was T4NXM1, UICC stage IV. After surgery, the patient was treated with gemcitabine. Although his activities of daily living had improved at the beginning of chemotherapy, he died five months after surgery due to progression of the cancer.

The histological diagnosis in both cases was determined as the pleomorphic type of anaplastic pancreatic cancer because tissue imaging with hematoxylin and eosin (H&E) staining was composed of a large portion of pleomorphic cells and a portion of spindle cells and poorly differentiated adenocarcinoma in the pancreas.

### Establishment of cell lines and cell culture

Samples of the patients’ ascites were removed in CELLSTAR® tubes (Greiner Bio-One GmbH, Frickenhausen, Germany) and centrifuged at 1500 rpm for 5 min. The pellets were suspended in Dulbecco’s Modified Eagle’s Medium (DMEM; Wako, Osaka, Japan) containing 10 % heat-inactivated fetal bovine serum (FBS; Nichirei Biosciences, Tokyo, Japan), 100 IUmL^−1^ penicillin (Sigma, Steinheim, Germany), 100 μgmL^−1^ streptomycin (Sigma), and 0.5 mM sodium pyruvate (Sigma). Then, the pellets were plated into a 100-mm culture dish (Falcon, Becton-Dickinson Labware, Lincoln Park, NJ, USA) and cultivated at 37 °C in a humidified atmosphere with 5 % CO2. The cell lines were trypsinized every 7–10 days, and maintained in complete culture medium. PDA cell lines (Panc-1, MIAPaCa-2, RWP-1, SW1990) and breast cancer cell line (MCF-7) were purchased from American Type Culture Collection (ATCC; Manassas, VA, USA) for use in various assays. As above, these cell lines were also cultured and passed. The morphological findings on OCUP-A1 and OCUP-A2 were investigated by phase-contrast microscopy.

### Short tandem repeat (STR) genotyping

Short tandem repeat genotyping was performed using genomic DNA extracted from OCUP-A1 and OCUP-A2. This analysis was performed by Promega (Tokyo, Japan). This experiment was conducted using the PowerPlex® 16 System (Promega, Madison, WI, USA) according to the manufacturer’s instructions.

### Karyotype analysis

G-banding was performed by SRL, Inc. (Tokyo, Japan) via the manufacturer’s procedures. The cells of OCUP-A1 and OCUP-A2 were cultured until exponential proliferation was reached for this analysis. Then, the cells were treated with colcemid (0.02 μg/mL) for 60 min and hypotonic treatment by potassium chloride (0.075 mol/L) for 20 min at 37 °C. The cells were fixed by Carnoy’s solution (methanol: acetic acid = 3: 1 by volume) for 10 min. The slides were air-dried and analyzed via G-banding. G-banding was carried out by trypsinizing the slides and stained by Giemsa staining using laboratory procedures. The slides were analyzed with the iCas system (Flovel Company, Ltd., Tokyo, Japan), and the images of the metaphases were captured. Using approximately 50 cells in both cell lines, the number of chromosomes was counted. Ten cells that resembled the mode number were karyotyped in detail.

### DNA extraction

Genomic DNA samples of OCUP-A1 and OCUP-A2 were extracted using the QIAamp® DNA Mini Kit (Qiagen, Venlo, Netherlands) according to the manufacturer’s protocol. The NanoDrop system (Invitrogen, Carlsbad, CA, USA) quantified the extracted DNA and estimated the quality before sequencing.

### Deep sequencing using Ion AmpliSeq™ cancer hotspot panel v2

The Ion AmpliSeq™ Cancer Hotspot Panel v2 (Life Technologies, Carlsbad, CA, USA) was used as multigene panels for sequencing. The amplicon library for exploring hotspot mutations of 50 cancer-related genes was generated using 10 ng of DNA from each sample. The 50 genes were as follows: ABL1, AKT1, ALK, APC, ATM, BRAF, CDH1, CDKN2A, CSF1R, CTNNB1, EGFR, ERBB2, ERBB4, EZH2, FBXW7, FGFR1, FGFR2, FGFR3, FLT3, GNA11, GNAS, GNAQ, HNF1A, HRAS, IDH1, JAK2, JAK3, IDH2, KDR/VEGFR2, KIT, KRAS, MET, MLH1, MPL, NOTCH1, NPM1, NRAS, PDGFRA, PIK3CA, PTEN, PTPN11, RB1, RET, SMAD4, SMARCB1, SMO, SRC, STK11, TP53, and VHL. Multiplex polymerase chain reaction amplification was conducted with 10 ng of DNA samples to make the sequence library according to the Ion AmpliSeq™ Library Preparation kit (Rev. A. 0 Jan 2014, Life Technologies). The quality of the library was estimated by the Agilent 2100 Bioanalyzer (Agilent Technologies, Santa Clara, CA, USA). After emulsion PCR, sequencing was run on the Ion PGM™ system (Life Technologies). The Torrent Suite™ software program v 4.0.2 (Life Technologies) was used to analyze the data, including alignment of the sequences to the reference genome (human genome build 19) and base calling. Variants were detected by the Torrent Variant Caller plug-in v 4.0-r76860 (Life Technologies) and visualized by the Integrative Genomics Viewer (Broad Institute, Cambridge, MA, USA). These procedures were consigned to TaKaRa Bio, Inc. (Otsu, Shiga, Japan). For a reliable sequence variant, a sequencing coverage of 250× and a variant frequency of at least 10 % in the background of the wild type were used as minimum requirements in the current study, as well as in the previous study [12].

### Proliferation

For estimation of proliferation, the doubling times of OCUP-A1, OCUP-A2 and 4 PDA cell lines were measured. A 6-well plate was used to culture these cell lines. There were 1.5 × 10^5^ cells per well that were disseminated and incubated at 37 °C in a humidified atmosphere with 5 % CO2. When the cell number count was performed, monolayer cells were trypsinized. The cell number was counted every 24 until 96 h after the beginning of culture. The Bio-Rad TM10™ automated cell counter (Bio-Rad Laboratories, Hercules, CA, USA) was used. The doubling time was determined from the growth curve obtained by the count.

### Migration and invasion

The migratory and invasive abilities were estimated using the IncuCyte ZOOM (Essen BioScience, Tokyo, Japan). These assays were performed according to the manufacturer’s protocol. Before assay, each pancreatic cancer cell line was cultured in a 96-well plate, and incubated until semi-confluent in 5 % CO2 at 37 °C. Then, the 96-pin WoundMaker™ (Essen BioScience) created a wound in each well of the 96-well plate. After washing by phosphate buffered saline (PBS), DMEM + 2%FBS was added into each well, and the plate was placed into the IncuCyte. The scratched area was scanned with the IncuCyte™ Live-Cell Imaging System and software (Essen BioScience) every 3 h. With the invasion assay, each pancreatic cancer cell line was cultured on Matrigel® (Corning, Inc, Corning, NY, USA) in a 96-well plate then incubated until semi-confluent in 5 % CO2 at 37 °C. After washing with PBS, DMEM + 2%FBS and Matrigel® of the same quantity as the medium were added. Similarly to the migration assay, the scratched area was scanned with the IncuCyte™ Live-Cell Imaging System and software (Essen BioScience) every 3 h.

### Chemosensitivity

To estimate the effect of anti-cancer drugs on the viability of the 6 pancreatic cancer cell lines, a 3- (4, 5- dimethylthiazol-2-yl) - 2, 5- diphenyltetrazolium bromide (MTT, Wako, Osaka, Japan) colorimetric assay was performed. The cancer cells (5 × 10^3^ cells/well) were seeded into a 96-well plate in DMEM. After 24 h, a different concentration of anti-cancer drugs was added to each well. Furthermore, after incubation for 72 h at 37 °C, 10 μL of MTT (5 mg/mL in PBS) were added to each well and the plates were incubated at 37 °C for 3 h, and then 200 mL of dimethyl sulfoxide (Wako) was added. The formazan product of MTT was measured as the absorbance at 550 nm using a microtiter plate leader (Model 550; Bio-Rad Laboratories, Tokyo, Japan). The percentage of cell viability was determined as the ratio of the absorbance of the sample to control. The IC_50_ value was measured as the drug concentration showing 50 % cell growth inhibition compared with the control cell proliferation in the 6 cell lines. The anti-cancer drugs used were 5-fluorouracil (5-FU; Kyowa Hakko Kirin Co., Ltd., Tokyo, Japan), gemcitabine (GEM; Yakult Honsha Co., Ltd., Tokyo, Japan), irinotecan (IRI; Yakult Honsha), oxaliplatin (OXA; Yakult Honsha), and paclitaxel (PTX; Nippon Kayaku Co., Ltd., Tokyo, Japan).

### Tumor markers and VEGF secreted from cell lines

There were 10^6^ cells of each cell line that were cultured in 10 mL of DMEM + 5%FBS for 4 days. Each supernatant was used for measuring the levels of CEA, CA19-9, SPan-1, DUPAN-2, and vascular endothelial growth factor (VEGF). These 4 tumor markers are generally measured for PDA patients. CEA was estimated by immunoassay (ARCHITECT® CEA, Abbott Japan, Tokyo, Japan). CA19-9 was investigated by chemiluminescent enzyme immunoassay (Kemirumi ACS-CA19-9 II, Siemens Japan, Tokyo, Japan). SPan-1 was measured by immunoradiometric assay (SPan-1 RIA Beads, TFB, Inc, Tokyo, Japan). DUPAN-2 and VEGF were investigated by enzyme-linked immunosorbent assay (ELISA) (DUPAN-2: Detamina-DUPAN-2, Kyowa Medex Co., Ltd., Tokyo, Japan; VEGF: Human VEGF Quantikine ELISA Kit, R&D Systems, Minneapolis, MN, USA). These analyses were conducted according to each manufacturer’s method by the Mitsubishi Chemical Medience Corporation, Tokyo, Japan.

### Angiogenesis (tube formation) assay

Angiogenesis affected by cancer cells was investigated using the CellPlayer™ Angiogenesis PrimeKit (Essen BioScience). This assay was performed according to the manufacturer’s method. Before starting the assay, 10^6^ cells of each cell line were cultured in 10 mL of DMEM + 5%FBS for 4 days. The supernatant was added to co-cultured green fluorescent protein-labeled human umbilical vein endothelial cells (HUVECs) and normal human dermal fibroblasts in a 96-well plate, and then HUVEC tube formation was monitored using IncuCyte ZOOM (Essen BioScience). As negative and positive control of angiogenesis, 5%DMEM and VEGF (4 ng/mL) were used instead of the supernatant of each cell line, respectively. The medium was replaced on days 5, 7, and 9 after cell plating. Phase-contrast and fluorescent images of tube formation were automatically captured, and tube length and branch points were also automatically measured every 6 h for 10 days using IncuCyte ZOOM (Essen BioScience). Kinetic plots of the angiogenesis metrics (tube length, tube area, and branch points) could be formed using the IncuCyte software.

### Proliferation under hypoxia

For investigating the proliferation of OCUP-A1 and OCUP-A2 under low oxygen condition, the growth of each cell line was compared between normoxia and hypoxia. Cancer cells (5 × 10^3^ cells/well) in DMEM + 10%FBS were seeded into a 96-well plate and incubated at 37 °C in a humidified atmosphere with 5 % CO2 and 1 or 21 % O2. Using IncuCyte ZOOM (Essen Bio-Science), two images per well were captured in both phase-contrast and fluorescence every 3 h during 48 h. The proportions of the fluorescent areas to all areas in the images were measured by the software under both hypoxia and normoxia. The fluorescent areas were regarded as indicating cell growth. Then, the cell viability under hypoxia at 48 h was calculated considering the cell viability under normoxia at 48 h as control in each cell line.

### Western blotting

Proteins expressions of E-cadherin (epithelial marker) and vimentin (mesenchymal marker) of OCUP-A1 and OCUP-A2 were examined by Western blotting. Approximately 30 μg of protein extracts were separated through 10 % sodium dodecyl sulfate polyacrylamide gel electrophoresis (SDS-PAGE) and transferred onto a polyvinylidene difluoride membrane using the Trans- Blot® Turbo™ Transfer System (Bio-Rad Laboratories, Inc., Hercules, CA, USA). Then, using the SNAP i.d.® 2.0 system (Merck Millipore, Darmstadt, Germany), the membranes underwent application of Tris-buffered saline-Tween (TBS-T) solution containing each primary antibody against anti-rabbit E-cadherin (1:300, Cell Signaling Technology, Danvers, MA, USA), anti-rabbit vimentin (1:300, Cell Signaling Technology), and anti-mouse β-actin (1:2500, Sigma-Aldrich, St. Louis, MO, USA) for 10 min after blocking with ECL blocking agent (GE Healthcare Life Sciences, Little Chalfont, Buckinghamshire, UK) and incubated with HRP-conjugated anti-mouse or anti-rabbit secondary antibody (1:5000, Sigma-Aldrich) for 10 min. Protein bands were visualized with the Luminescent Image Analyzer LAS 4000-plus (Fuji, Tokyo, Japan).

### Side population (SP) analysis using flow cytometry

Stem-like cells were measured by SP analysis. The cells were incubated in pre-warmed DMEM supplemented with 5 % FBS containing 5 μg/mL Hoechst 33342 (Sigma Chemicals, St Louis, MO, USA) in the absence or presence of 30 μg/mL verapamil at 37 °C for 60 min. Then, 1 μg/mL propidium iodide was added and then filtered through a 40-μm cell strainer (Becton Dickinson, San Diego, CA, USA) and maintained at 4 °C. The analysis was performed using FACS AriaII (Becton Dickinson). Hoechst 33342 was excited with the UV laser at 350 nm, and fluorescence emission was measured with 405/BP30 (Hoechst blue) and 570/BP20 (Hoechst red) optical filters.

### Tumorigenicity in vivo

There were 10^7^ viable cells of each cell line (OCUP-A1, OCUP-A2, Panc-1, and MIAPaCa-2) that were suspended by 200 μL of DMEM + 10%FBS and were inoculated subcutaneously into the back of four-week-old BALB/c nude mice. In each cell line, five mice were injected subcutaneously. In all cases, the injected cells developed a subcutaneous tumor. During approximately one month, the diameter of the subcutaneous tumor in each mouse was measured every 3–5 days. The volume of the tumor was calculated with the formula:

$$ \mathrm{Tumor}\ \mathrm{volume}\ \left({\mathrm{mm}}^3\right) = \left(\mathrm{long}\ \mathrm{diameter}\ \left(\mathrm{mm}\right)\right) \times {\left(\mathrm{short}\ \mathrm{diameter}\ \left(\mathrm{mm}\right)\right)}^2 \times 0.5 $$

The mice were sacrificed with an overdose of sevoflurane. Part of each subcutaneous tumor was fixed in 10 % formalin and embedded in paraffin section for staining and immunohistochemistry.

### Immunohistochemistry

Tissues from primary APC were obtained from the 2 study patients who underwent surgery at our institution. Then, the xenografts of OCUP-A1 and OCUP-A2 were also used for immunohistochemistry. Formalin-fixed paraffin-embedded specimens were made from each tissue sample. In each sample, the representative block of APC and the cell lines were chosen. Each block was sliced at 4 μm for H&E staining and immunohistochemistry for E-cadherin and vimentin. Immunohistochemistry was performed according to the protocol of our institution. We used antibodies against E-cadherin (1: 200, Abcam, Cambridge, UK) and vimentin (1: 200, Dako, Glostrup, Denmark) for immunohistochemical labeling. The expression of each protein was scored as 0 (no labeling in cancer cells), 1+ (mild labeling), 2+ (moderate labeling), and 3+ (strong labeling). The scoring was performed by two surgeons and a pathologist.

### Statistical analysis

Results of statistical analyses were expressed as the means ± standard error from at least three independent experiments. Significance of difference was analyzed with Student’s t tests or Tukey’s honest significant difference (HSD) test using JMP 10 (SAS Institute, Inc., Cary, NC, USA). P-values less than 0.05 were regarded as statistically significant.

## Results

### Morphology of new established cell lines, OCUP- A1 and OCUP- A2

**Fig. 1:**
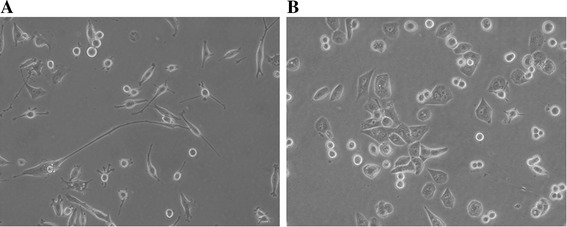
Photographs of the two established anaplastic pancreatic cancer cell lines taken by the phase-contrast microscope. **a** OCUP-A1. (original magnification × 100). **b** OCUP-A2. (original magnification × 100)

We succeeded in establishing the cell lines of OCUP- A1 and OCUP- A2. Although both cell lines had little adhesion in a non-confluent state, they formed monolayer sheets in confluence. The cells of OCUP- A1 were mainly spindle-shaped, and appeared as if they were extended for communication among the cancer cells (Fig. 1a). In addition, a few polygonal and large cells were observed in the cell line. Many cells of OCUP- A2 were polygonal and formed clusters by adhesion among the cancer cells. In addition, a few round cells were observed in OCUP- A2 (Fig. 1b).

### STR assay

All DNA extracted from the 2 established cell lines showed identical STRs, which did not corresponding to the cells of the database of the Japanese Collection of Research Bioresources (JCRB) (Database of 2279 cells registered in the ATCC, the Deutsche Sammlung von Mikroorganismen und Zellkulturen, and the Japanese Collection of Research Bioresources).

### Chromosome analysis

**Fig. 2:**
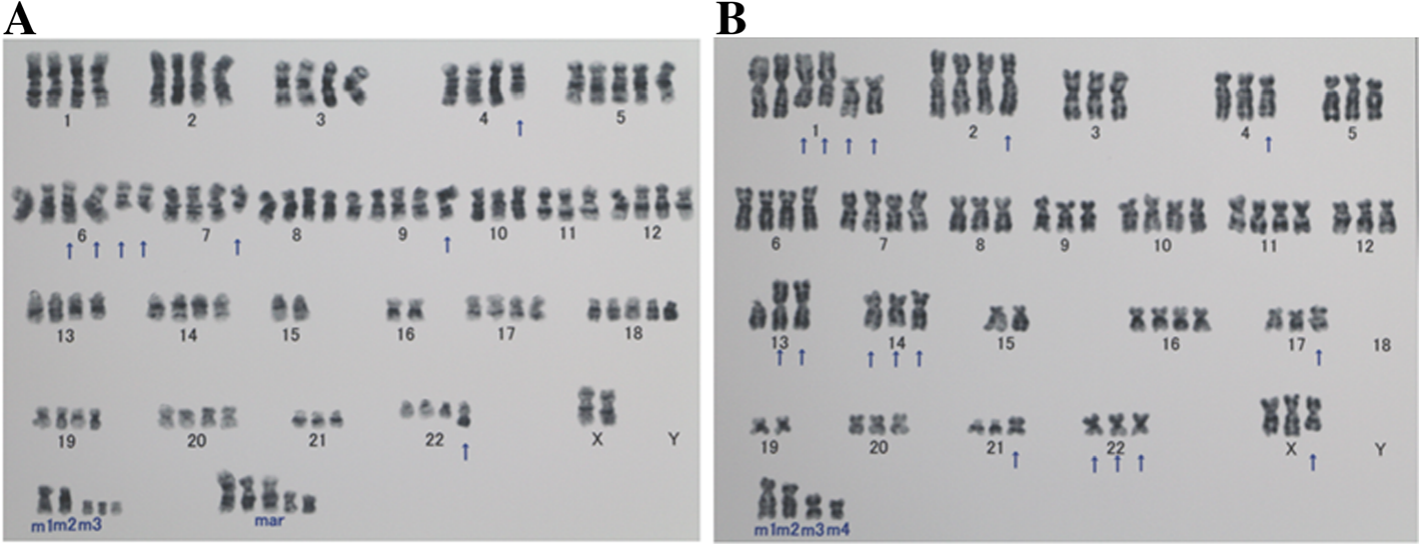
Representative karyotype analysis of the two established anaplastic pancreatic cancer cell lines using the G-banding method. The figure displays the chromosome construction of OCUP-A1 (Fig. 2a) and OCUP-A2 (Fig. 2b). The arrow designates the reconstructive chromosome. The marker chromosome is different from standard diploid and has a break point

The chromosome number of OCUP- A1 ranged from 55 to 106. The range of chromosome numbers of OCUP- A2 was 74 to 79. Figure 2 shows a representative karyotype of both cell lines. Both cell lines had many chromosomal abnormalities in the number and structure. An abnormality in OCUP- A1 was identified: add(4) (q21), +5, +add(6) (q13) x2, del(6) (q?) x2,del(7) (q22) (q13), −10, −11, −15, −15, −16, −16, +18, −21, add(22) (q13), +mar1, +mar2, +mar3x3, +5mar. A chromosomal abnormality in OCUP- A2 was: +del(X) (q?), +1, +der(1;15) (p10;q10) x2, der(1;19) (q10;p10), der(1;19) (q10;p10), der(2) t(2;7) (p11.2;q11.2), del(4) (q?), +6, +7, +10, +11, add(13) (p11.2) x2, add(14) (p11.2) x2, der(14;18) (q10;q10), −15, +16, add(17) (p11.2)x2, −18, −18, +19, add(21) (p11.2), add(22) (p11.2)x3, +mar1, +mar2, +mar3, +mar4.

### Gene analysis

A sufficient library from each sample led to subsequent successful sequencing. In DNA samples of OCUP-A1 and OCUP-A2, a mean 100× coverage of 98.8 % and 97.3 % with a mean read length of 105 bp was obtained, respectively.

Thirteen gene mutations of the targeted genes were identified in both cell lines (Table 1). 9 gene mutations (ERBB4, FGFR3, PDGFRA, KDR, APC, KRAS, FLT3, TP53, and STK11) were common to each cell line. The sequence variations of these mutations were all single nucleotide polymorphisms, except for one of the TP53 mutations identified in OCUP-A1. KRAS mutations were in codon 12 in both samples. TP53 mutations consisted of various variations including three novel mutations: g.7578222_7578225del, g. 7578369 A > C, and g. 7579472 G > C. Most of the other detected mutations were newly identified, whereas the mutations of PIK3CA and CSF1R were detected only in OCUP-A1. Similarly, the mutations of ALK and RET were identified only in OCUP-A2.

**Table 1:** Gene mutation status of OCUP-A1 and OCUP-A2

Chromosome #	Gene	OCUP-A1	OCUP-A2
2	ERBB4	g. 212812097 T > C (54.2 %)	g. 212812097 T > C (18.4 %)
2	ALK	-	g. 29432625 C > A (16.4 %)
3	PIK3CA	H1047R (77.3 %)	-
4	FGFR3	g. 1807894G > A (100 %)	g. 1807894G > A (100 %)
4	PDGFRA	V824V (97.5 %)	g. 55141055 A > G (100 %)
		g. 55141055 A > G (100 %)	
4	KDR	g. 55980239 C > T (99.0 %)	g. 55980239 C > T (100 %)
5	APC	g. 112175770 G > A (97.7 %)	g. 112175770 G > A (96.9 %)
5	CSF1R	g. 149433596 G > A (99.5 %)	-
		g. 149433597 C > T (95.3 %)	
10	RET	-	g. 43613843 G > T (63.5 %)
12	KRAS	G12D (98.5 %)	G12R (57.3 %)
13	FLT3	g. 28610183 A > G (100 %)	g. 28610183 A > G (100 %)
17	TP53	R209fs*6 (50.3 %) D208E (99.6 %)	g. 7578369 A > C (100 %)
		g.7578222_7578225del (49.7 %)	g. 7579472 G > C (92.3 %)
19	STK11	g. 1220321 T > C (42.0 %)	g. 1220321 T > C (100 %)

Gene mutations were examined with Ion AmpliSeq™ Cancer Hotspot Panel v2

### Proliferation

Table 2 shows the doubling times of the 6 cell lines including OCUP- A1 and OCUP- A2. The doubling times of OCUP-A1 and OCUP-A2 did not have prominently short doubling times compared to other PDA cell lines.

**Table 2:** Doubling time in pancreatic cancer cell lines

Cell line	Doubling time (hours)
OCUP-A1	20.6 ± 1.2
OCUP-A2	20.6 ± 1.4
Panc-1	25.8 ± 2.8
MIAPaCa-2	25.7 ± 4.3
RWP-1	23.6 ± 0.54
SW1990	35.8 ± 4.4

OCUP-A1 and OCUP-A2 vs SW1990; *p* < 0.05

### Migration assay and invasion assay

**Fig. 3:**
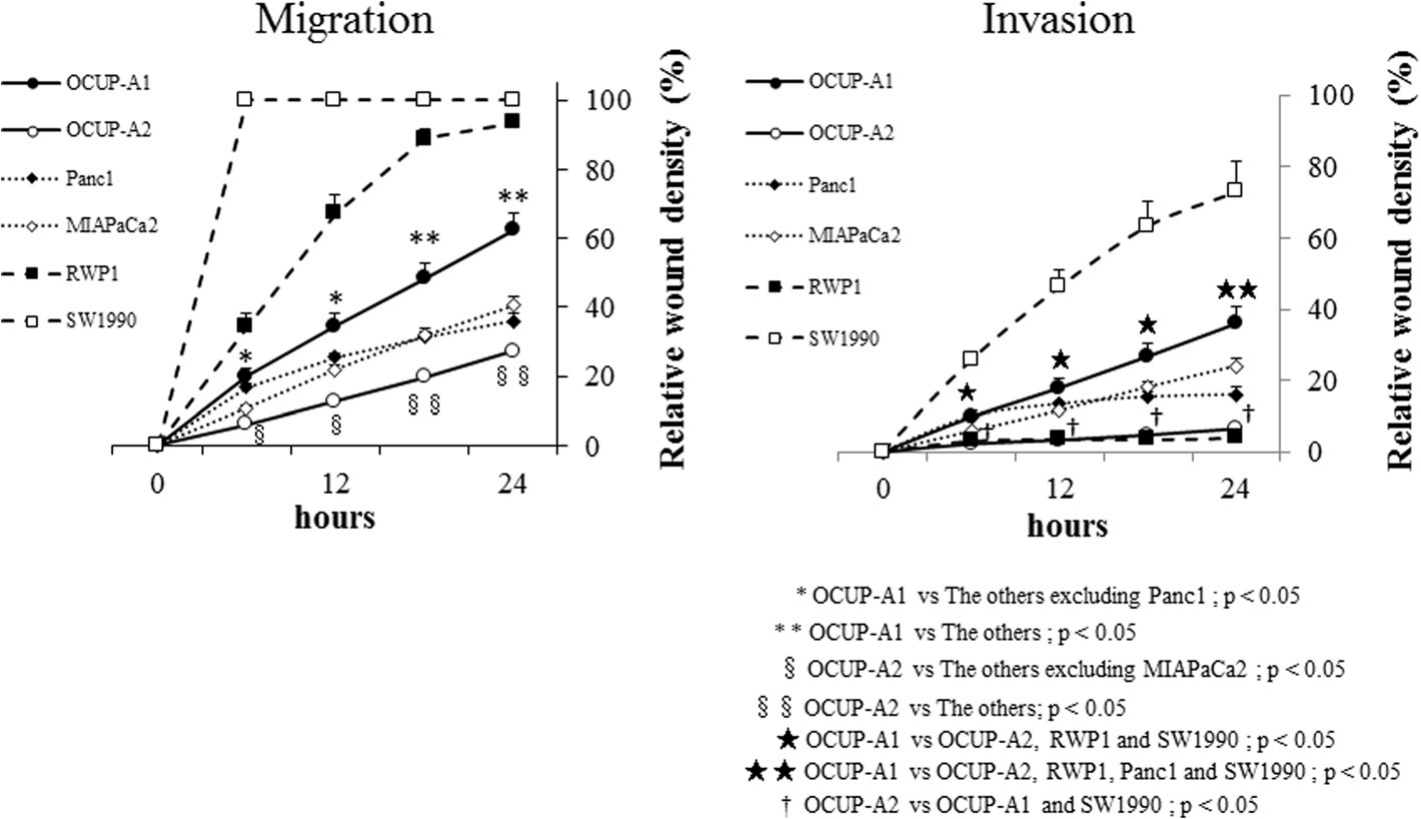
Migration and invasion of pancreatic cancer cell lines. Relative wound density designates the percentage of scratch area where cancer cells migrated or invaded

Figure 3 shows the wound closure proportion (relative wound density; RWD) as migration or invasion abilities of each cell line every 3 h from starting the test to 24 h. In the migration assay, the RWD at 24 h of OCUP-A1 was significantly superior to that of OCUP-A2, Panc1 and MIAPaCa2, but inferior to that of RWP1 and SW1990. Then, the RWD at 24 h of OCUP-A2 was significantly lowest in all 6 cell lines. In the invasion assay, the RWD at 24 h of OCUP-A1 was significantly superior to that of OCUP-A2, RWP1 and Panc1, but inferior to that of SW1990. The RWD at 24 h of OCUP-A2 was significantly lower than that of OCUP-A1 and SW1990.

### Drug sensitivity

The IC_50_ values of each cell line are shown for each anti-carcinogenic agent in Table 3. Both of OCUP-A1 and OCUP-A2 showed relatively high chemosensitivity to GEM. OCUP-A1 showed relatively low sensitivity to 5-FU. OCUP-A2 showed the highest sensitivities to IRI and OXA among 6 cell lines tested. OCUP-A2 was also relatively sensitive to 5-FU and PTX.

**Table 3:** IC_50_ values of anti-cancer drugs for pancreatic cancer cell lines

Drug	5-FU (μM)	GEM (nM)	IRI (μM)	OXA (μM)	PTX (nM)
OCUP-A1	51.1 ± 5.1	6.60 ± 1.4	5.21 ± 0.20	17.6 ± 0.94	15.7 ± 0.60
OCUP-A2	23.8 ± 6.3	6.01 ± 0.60	1.67 ± 0.075*	1.63 ± 0.070*	3.63 ± 0.14*
Panc-1	24.2 ± 12	59.2 ± 5.5***	8.90 ± 0.49***	9.25 ± 1.4	27.8 ± 0.95***
MIAPaCa-2	10.7 ± 2.4*	24.0 ± 10***	15.3 ± 0.28***	20.1 ± 0.91**	6.92 ± 0.14*^,^**
RWP-1	11.6 ± 1.8*	4.45 ± 1.3	7.02 ± 0.50***	18.4 ± 0.88	3.45 ± 0.046*
SW1990	68.2 ± 15**	92.0 ± 4.3***	10.5 ± 0.47***	72.9 ± 6.8***	21.3 ± 0.86***

*5-FU* 5-fluorouracil, *GEM* gemcitabine, *IRI* irinotecan, *OXA* oxaliplatin, *PTX* paclitaxel

*vs OCUP-A1; *p* < 0.05

**vs OCUP-A2; *p* < 0.05

***vs OCUP-A1 and OCUP-A2; *p* < 0.05

### Tumor markers secreted from cell lines

The values of tumor markers in the supernatant of the cell lines are shown in Table 4. OCUP-A1 and OCUP-A2 did not much secrete any of tumor markers.

**Table 4:** Tumor markers in supernatant of pancreatic cancer cell lines

	CEA	CA19-9	SPan-1	DUPAN-2
	(ng/mL)	(U/mL)	(U/mL)	(U/mL)
OCUP-A1	<0.5	<1.2	<3	<25
OCUP-A2	<0.5	<1.2	<3	<25
Panc-1	<0.5	<1.2	<3	<25
MIAPaCa2	<0.5	<1.2	<3	<25
RWP1	24.6	3020	865	24.6
SW1990	2.8	4720	2050	2.8

*CEA* carcinoembryonic antigen, *CA19-9* carbohydrate antigen 19-9

### Angiogenesis and VEGF secreted from cell lines

**Fig. 4:**
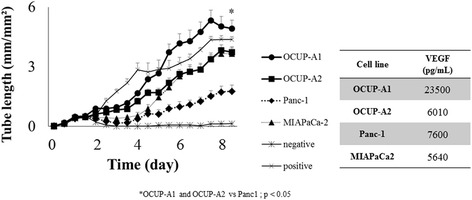
Tube formation of human umbilical vein endothelial cells with vascular endothelial growth factor (VEGF) stimulation derived from cell lines and the values of VEGF in supernatant of pancreatic cancer cell lines

As seen in Fig. 4, HUVEC showed cord extension according to the addition of supernatant of cultured cancer cells or VEGF control. The cord extension of each sample was evaluated using tube length per unit area, indicating induction of angiogenesis. The supernatant of all cell lines gradually caused tube extension of HUVEC. At day 8, the supernatant of OCUP-A1, OCUP-A2, Panc1, MIAPaCa2 and positive control (VEGF administration) extended the tube length to 5.16 ± 0.23, 3.75 ± 0.070, 1.77 ± 0.16, 3.58 ± 0.33 and 4.33 ± 0.052 mm/mm^2^, respectively. However, negative control (FBS included in the supernatant) hardly caused cord extension (tube length at day 8; 0.14 ± 0.019 mm/mm^2^). The culture supernatant of OCUP-A1 and OCUP-A2 significantly contributed to longer cord extension than Panc-1. The value of VEGF in the supernatant of each cell line were shown in Fig. 4. All cell lines secreted VEGF, and OCUP- A1 showed the maximum secretion of VEGF among the 4 cell lines.

### Proliferation under hypoxia

**Fig. 5:**
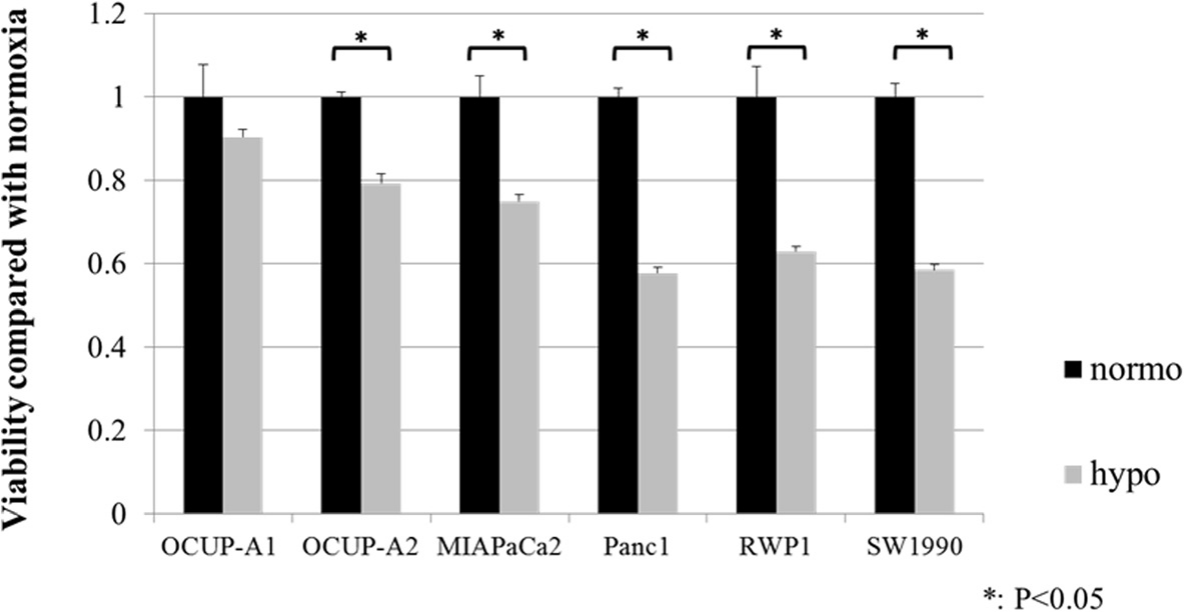
Regarding confluence incubated under hypoxic condition as standard value, the confluence under hypoxic condition was compared with the confluence under normoxic condition in each cell line

Figure 5 shows that OCUP- A1 was the only cell line that did not significantly change proliferation between hypoxia and normoxia. The proliferation of OCUP-A2 significantly decreased by approximately 20 % under hypoxia compared to normoxia. And the proliferations under hypoxia of other cell lines also significantly decreased by about 25 to 40 % compared to under normoxia.

### Western blotting

**Fig. 6:**
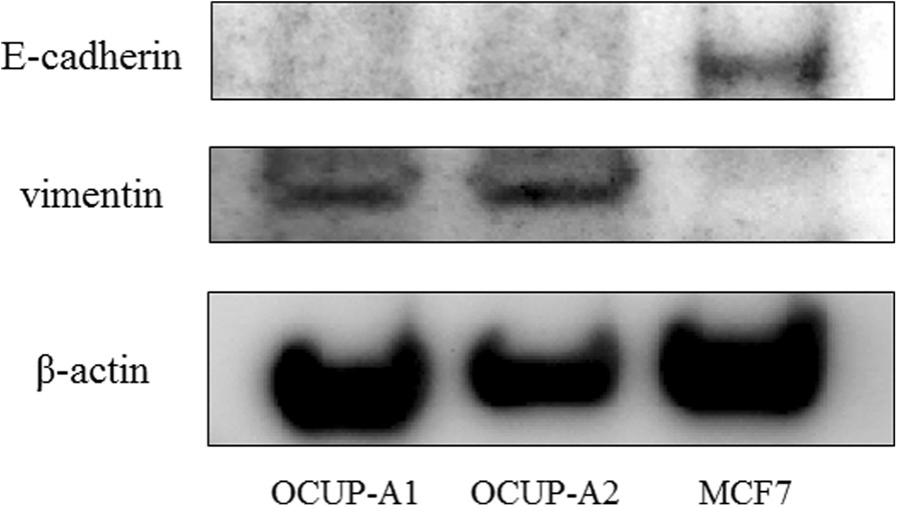
Western blot analysis of E-cadherin and vimentin of OCUP-A1 and OCUP-A2. MCF7 was used as positive E-cadherin and negative vimentin controls

The protein bands of OCUP-A1 and OCUP-A2 were compared with that of the breast cancer cell line MCF7, which was known to expresses E-cadherin (Fig. 6). Both OCUP-A1 and OCUP-A2 expressed vimentin but did not express E-cadherin, while MCF7 showed the expression of E-cadherin and the absence of vimentin.

### SP cell analysis

**Fig. 7:**
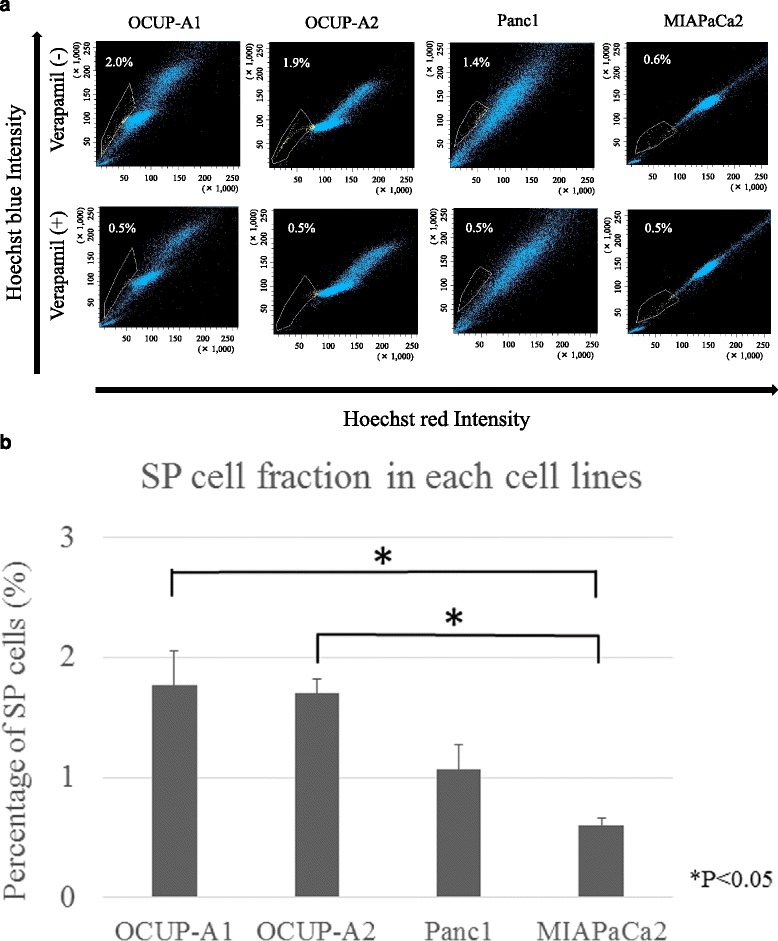
Analysis of side population (SP) cells in pancreatic cancer cell lines. **a** Representative figure of flow cytometry. The fraction of SP cells in each cell line is outlined by the white line. Each cell line was stained with Hoechst 33342 in the presence or absence of verapamil (shown as bottom panel or upper panel, respectively). SP cells disappeared with verapamil. **b** The percentage of SP fraction in each cell line

Figure 7a is a representative picture of flow cytometric analysis. The fraction of SP cells is enclosed by the white line (upper panel) and disappeared under the presence of verapamil (bottom panel). The percentage of the total cells is shown close to the white line. The average SP percentages of OCUP-A1, OCUP-A2, Panc-1 and MIAPaCa2 were 1.8 ± 0.28 %, 1.7 ± 0.12 %, 1.1 ± 0.20 % and 0.6 ± 0.058 %, respectively (Fig. 7b). The proportion of SP cells in OCUP-A1 and OCUP-A2 was significantly higher than that in MIAPaCa2.

### Tumorigenicity of OCUP- A1 and OCUP- A2 in nude mice

**Fig. 8:**
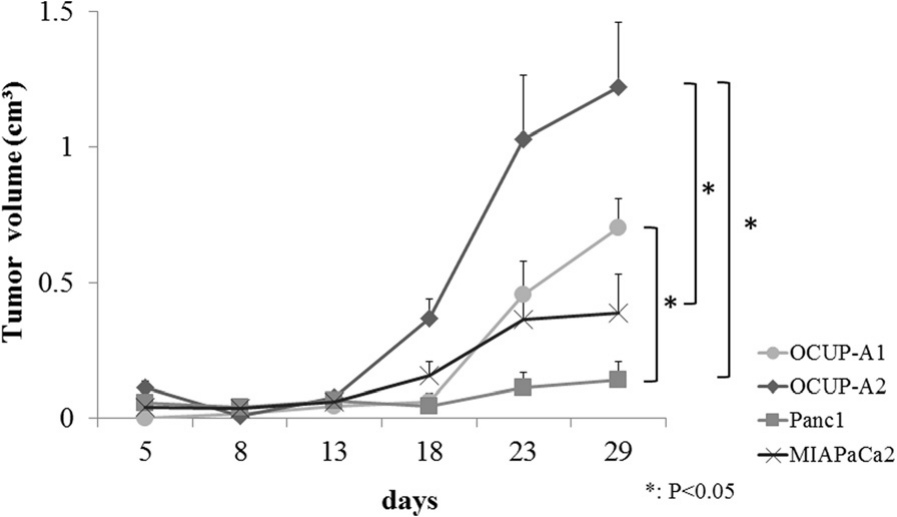
Growth curve of xenografts of pancreatic cancer cell lines (*n* = 5). Tumors developed in all mice using for this assay

In all cell lines, xenografts were successfully made from all mice. Figure 8 shows the growth curve of the xenografts of the four cell lines. At 29 days after injection, the tumor volume of the xenograft of OCUP-A2 increased significantly more than that of Panc-1 and MIAPaCa2. The tumor size of the mice given an injection of OCUP-A1 was also significantly larger than that of Panc-1. The volume did not statistically differ between OCUP-A1 and OCUP-A2.

### Immunohistochemical staining of epithelial-mesenchymal transition (EMT)-related markers

**Fig. 9:**
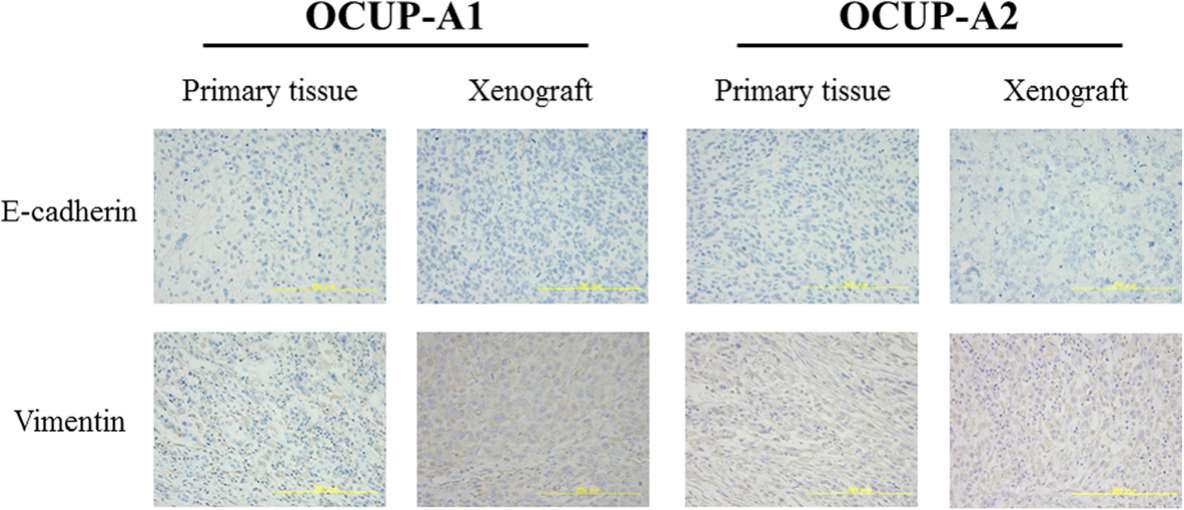
Immunostaining of primary tumor and xenograft in OCUP-A1 and OCUP-A2. All specimens showed loss of E-cadherin expression and gain in vimentin expression. The expression pattern was consistent with the epithelial-mesenchymal transition (EMT) phenotype (original magnification × 400)

Immunohistochemical stainings of the primary tumor and the xenograft of OCUP-A1 and OCUP-A2 were negative for E-cadherin, but almost positive for vimentin (Fig. 9). The expression patterns of these proteins in OCUP-A1 and OCUP-A2 were similar to those in western blotting.

## Discussion

Due to the rarity, the characteristics of APC are not well documented. The present study intended to clarify the biological behaviors of APC using two novel APC cell lines established in our institute, compared to conventional PDA. It may create an important role for future pancreatic therapy.

Conventional chromosomal analysis has drawn attention to significant implications of common chromosomal aberrations in pancreatic cancer [13]. The chromosomal analyses performed in the present study identified numerical and unbalanced structural abnormalities in both cell lines such as loss of chromosome Y or/and chromosome 18, which are frequently seen in pancreatic cancer [13]. However, the chromosomal aberrations of these two cell lines did not correspond to chromosomal aberrations of other APC samples [14]. The various aberrations in APC might demonstrate the heterogeneity and biological complexity of APC.

Regarding gene mutation analysis, there were common and different features between APC and PDA. Mutations of KRAS and TP53 were identified in OCUP-A1 and OCUP-A2. Although these mutations have been reported most frequently in ordinary PDA [15, 16] and in APC [6, 7], the SMAD4 mutation that was also observed in PDA [15, 16] was not detected in both cell lines. It is unclear whether the established cell lines only happened to be composed of cells that had no mutation of SMAD4, or whether APC itself expresses the wild type of the gene; these mutations have seldom been reported in APC, unlike the mutations of KRAS and TP53. As a mutation of low frequency in PDA, the STK11 mutation [2, 16, 17] was also found in both cell lines. In addition, although we detected genetic alterations of the genes STK11 and APC (adenomatous polyposis coli), which are related to genetic syndromes associated with familial pancreatic cancer such as Peutz-Jeghers syndrome or familial adenomatous polyposis [17], it is unknown whether the mutations were germline mutations because of not investigating the genes in their normal tissues. From the current sequencing, it is suggested that the progression of APC might be related to many complicated mutations.

Several assays were performed for investigating the aggressiveness of APC using the experimental model in the present study. The results demonstrated that APC cell lines did not have prominent abilities of proliferation, migration and invasion compared to other PDA cell lines in vitro. However, the tumorigenicity in vivo of OCUP-A2 was stronger than that of MiaPaca-2 and Panc-1 and that of OCUP-A1 was also stronger than that of Panc-1. These results were consistent with its clinical characteristics that APC often develops huge tumor. The average size in most series of APC has been reported to be 9 or 10 cm. [8]

We hypothesized that the high proliferation ability in vivo and in clinical observation might arise from the tumor microenvironment and increment of stem-like cells. Therefore, we performed assays for the angiogenesis, hypoxic tolerance and SP cells. The cord formation of HUVEC and production of VEGF showed that angiogenesis of OCUP-A1 was superior to that of other PDA cell lines resulting from the higher production of VEGF. Previous studies have suggested that VEGF and its receptors (VEGFR) contribute to an autocrine/paracrine mitogenic loop via mitogen-activated protein kinase (MAPK) signaling on the proliferation of cancer cells, including pancreatic cancer, besides the stimulation of angiogenesis [18–20]. The secretion of VEGF from OCUP-A1 might play a role in active proliferation in vivo. Further examination is needed on whether VEGFRs are actually expressed on APC cell lines because we only observed a single nucleotide polymorphism (SNP) of KDR (VEGFR-2) on gene analysis.

The high malignancy of APC might also be related to hypoxia. The present study indicated that OCUP-A1 had hypoxia tolerance. Hypoxia tolerance could be formed through a gene mutation such as a p53 mutation, which relates to apoptosis, and the interaction between oncogenic signaling such as RAS and hypoxia-induced transcription factor (HIF) 1*α* [21]. It has also been reported that hypoxia enhances vascular endothelial cell growth [22]. Another previous report specifically showed that HIF-1 moderated VEGF transcription [23]. As a result, angiogenesis is induced by hypoxia, and then the above-mentioned autocrine mitogen for APC cells might be promoted, resulting in active proliferation in vivo and in clinical observation. In addition, it has been demonstrated that hypoxia is related to aggressive growth in pancreatic cancer using a xenograft model [24]. Future studies may be able to investigate whether hypoxia-induced aggressive growth correlates with the extent of gene mutation or VEGF expression in vivo.

SP cells in APC cell lines were more enriched than in MiaPaCa-2. Previous studies on SP analysis showed that SP cells related to tumorigenic potential and drug resistance, such as cancer stem cells in various type of tumors [25]. Especially in a pancreatic cancer cell line, to date, it has also been suggested that SP cells might contribute to aggressive tumor growth [26].

To sum up, these results suggested the biological aggressiveness of APC might be related with induction of angiogenesis, acquisition of tolerance to hypoxia, and increment of stem-like cells.

Chemosensitivity analysis showed that OCUP-A1 and OCUP-A2 were comparatively sensitive to several anti-cancer drugs including gemcitabine. OCUP-A2 was also the most sensitive to some of anti-cancer drugs among the examined cell lines. Strobel et al. reported that adjuvant or palliative chemotherapy did not contribute to improvement of prognosis in APC [3]. However, a different study demonstrated that paclitaxel treatment, which was selected by chemosensitivity examination, resulted in complete response to APC [10]. Furthermore, it has been reported that a subtype of PDA, which has highly expressed mesenchyme-associated genes, was more sensitive to gemcitabine than the classical type of this neoplasm [27]. The present study also suggested that selective use of anti-tumor drugs might even enable the aggressiveness of APC to be controlled.

Regarding EMT, the absence of E-cadherin and the expression of vimentin were observed in APC cell lines, xenografts and primary specimens from western blotting or immunohistochemistry. This is a feature of APC pathology [8, 28]. In pancreatic cancer, it has been reported that patients with absence of E-cadherin expression or the expression of vimentin had poorer prognosis than patients with the differentiated type [29, 30]. In addition, it has also been reported that the combination of these protein expression patterns was correlated with prognosis in pancreatic cancer patients [31]. In the present study, both patients from whom the two established cell lines were derived had very poor prognosis. OCUP-A1 and OCUP-A2 also reflected the high malignancy of primary tumor in terms of EMT status.

The present study was limited in two respects. One limitation was that these cell lines were samples derived from ascites. Therefore, OCUP-A1 and OCUP-A2 might lose the properties possessed in the primary site. Another limitation was the monoclonality of the cell lines OCUP-A1 and OCUP-A2 that could not reflect heterogenic properties of the pleomorphic type of APC. To resolve these limitations, a new APC cell line derived from a primary site needs to be established and then divided into several cell types using limiting dilution.

## Conclusion

The present study succeeded in establishing two APC cell lines. Angiogenesis, hypoxia tolerance, the presence of SP cells and EMT might be associated with the biology of APC. These cell lines could be useful for investigating the progression of pancreatic cancer. Effective treatment for APC could potentially be developed through further investigations using these cell lines.

### Ethics approval and consent to participate

This study was carried out in compliance with the Helsinki declaration and was approved by the ethics committee of Osaka City University. The consent was obtained from the two patients with APC to use ascites specimens and resected tissue samples of the two patients in writing. Also, the experiment about animals was approved by the Osaka City University Ethical Committee for animal experiments.

### Consent for publication

The consent to publish the individual data was obtained from the two patients in this study in writing.

## Abbreviations

5-FU: 5-fluorouracil
APC: anaplastic pancreatic cancer
ATCC: American type culture collection
CA19-9: cancer antigen 19-9
CEA: carcinoembryonic antigen
CT: computed tomography
DMEM: Dulbecco’s modified eagle’s medium
ELISA: enzyme-linked immunosorbent assay
EMT: epithelial-mesenchymal transition
FBS: fetal bovine serum
GEM: gemcitabine
H&E: hemotoxylin and eosin
HIF: hypoxia-induced transcription factor
HSD: honest significant difference
HUVECs: human umbilical vein endothelial cells
IRI: irinotecan
JCRB: Japanese collection of research bioresources
MAPK: Mitogen-activated protein kinase
MTT: 3- (4, 5- dimethylthiazol-2-yl) - 2, 5- diphenyltetrazolium bromide
OXA: oxaliplatin
PDA: pancreatic ductal adenocarcinoma
PTX: paclitaxel
RWD: relative wound density
SDS-PAGE: sodium dodecyl sulfate polyacrylamide gel electrophoresis
SNP: a single nucleotide polymorphism
SP: side population
STR: short tandem repeat
TBS-T: Tris-buffered saline-Tween
VEGF: vascular endothelial growth factor
VEGFR: vascular endothelial growth factor receptor
